# Simple In-Hospital Interventions to Reduce Door-to-CT Time in Acute Stroke

**DOI:** 10.1155/2016/1656212

**Published:** 2016-07-10

**Authors:** Elyar Sadeghi-Hokmabadi, Aliakbar Taheraghdam, Mazyar Hashemilar, Reza Rikhtegar, Kaveh Mehrvar, Mehrdad Mehrara, Reshad Mirnour, Rogayyeh Hassasi, Hannane Aliyar, Mohammadamin Farzi, Somayyeh Hasaneh Tamar

**Affiliations:** ^1^Neuroscience Research Center, Imam Reza Hospital, Tabriz University of Medical Science, Daneshgah Street, Tabriz, East Azerbaijan, Iran; ^2^Imam Reza Hospital, Tabriz University of Medical Science, Daneshgah Street, Tabriz, East Azerbaijan, Iran

## Abstract

*Background*. Intravenous tissue plasminogen activator, a time dependent therapy, can reduce the morbidity and mortality of acute ischemic stroke. This study was designed to assess the effect of simple in-hospital interventions on reducing door-to-CT (DTC) time and reaching door-to-needle (DTN) time of less than 60 minutes.* Methods*. Before any intervention, DTC time was recorded for 213 patients over a one-year period at our center. Five simple quality-improvement interventions were implemented, namely, call notification, prioritizing patients for CT scan, prioritizing patients for lab analysis, specifying a bed for acute stroke patients, and staff education. After intervention, over a course of 44 months, DTC time was recorded for 276 patients with the stroke code. Furthermore DTN time was recorded for 106 patients who were treated with IV thrombolytic therapy.* Results*. The median DTC time significantly decreased in the postintervention period comparing to the preintervention period [median (IQR); 20 (12–30) versus 75 (52.5–105), *P* < 0.001]. At the postintervention period, the median (IQR) DTN time was 55 (40–73) minutes and proportion of patients with DTN time less than 60 minutes was 62.4% (*P* < 0.001).* Conclusion*. Our interventions significantly reduced DTC time and resulted in an acceptable DTN time. These interventions are feasible in most hospitals and should be considered.

## 1. Introduction

In the setting of acute ischemic stroke, intravenous tissue plasminogen activator (IV-tPA), if given within three hours of symptoms onset, has been proven to reduce the combined endpoint of death and disability after stroke [1, 2]. However its benefit is strongly time dependent [3]. The American Heart Association/American Stroke Association (AHA/ASA) guideline emphasizes that suspected acute stroke patients should be triaged with the same priority as patients with acute myocardial infarction or serious trauma, regardless of the severity of neurological deficits. Based on these guidelines, the door-to-CT scan (DTC) time should be less than 25 minutes and door-to-needle (DTN) time should be less than 60 minutes [4]. For each 15-minute reduction in delay, there are an estimated 4% improvement in clinical outcome and 5% lower odds of mortality [5, 6]. Furthermore, recently some stroke professionals are calling for an aggressive update of these targets to a DTN benchmark of 30-minute median (60-minute 95th percentile) [7].

## 2. Aims

As reported elsewhere, at our hospital, the most important barriers to implement thrombolytic therapy for acute ischemic stroke patients were in-hospital delays such as initial patient assessment, performing CT scan, and lab studies [8, 9]. This quality-improvement study aimed to reduce in-hospital delay for acute stroke patients by putting simple interventions into practice which are mainly feasible in developing countries.

## 3. Methods

### 3.1. Design and Study Population

This study was set up as a before-versus-after study, divided into 2 periods: the preintervention period (21 April, 2009–20 April, 2010) for one year and the postintervention period (22 May, 2011–20 Jan, 2015) for 44 months. The study was performed at Tabriz Imam Reza Hospital, which is a tertiary referral university hospital in East Azerbaijan Province, Iran.

### 3.2. Definition of Time Intervals

We assessed the door-to-CT time (from emergency department arrival to performance of brain CT scan) and the door-to-needle time (from emergency department arrival to IV-tPA use).

#### 3.2.1. Preintervention Period

This was the pre-r-TPA stage in which thrombolysis was not available at our center for acute ischemic stroke patients. Any patient, who met the Cincinnati stroke scale, with sudden onset of at least one of the following symptoms (less than 3 hours of symptoms onset), was enrolled into the study: facial droop, motor arm weakness, or speech abnormalities. The demographic data and DTC time were recorded. However, not having r-TPA at the time, the DTN time could not be recorded. During this period, 213 patients were enrolled.

#### 3.2.2. Postintervention Period

This period is associated with the time when r-TPA was available in our center and some measures were already taken to improve in-hospital delays for treating stroke patients.

Any patient, for whom stroke code was activated, enrolled. Based on the algorithm, the code should be activated for patients with the onset of symptoms in previous three hours (according to Cincinnati stroke scale). Overall, the stroke code was activated for 321 patients. Given that during the preintervention period none of the patients had prehospital notification, forty-five patients in the postintervention period were excluded because they had prehospital notification. Finally, 276 patients enrolled in the postintervention period, in which IV r-TPA was administered in 106 patients. In addition to the demographic data and DTC time, DTN time was also recorded for these patients.

### 3.3. Intervention

Based on the results of the preintervention period, evaluation process of patients with probable stroke was too slow, so a multidisciplinary team consisting of two stroke neurologist, two attending physicians of emergency department (ED), neurology and ED residents, a neuro-ICU, and an ED nurse analyzed the existing process of approach to a probable acute stroke patient. At last, five feasible simple interventions were implemented, with a goal of enhancing the flow of patients and reducing in-hospital delay as follows.

*The Quality-Improvement Interventions*

1. Single call notification.
2. Prioritizing patient for CT scanning.
3. Prioritizing patient for lab analysis.
4. ICU bed.
5. Staff education.

**Figure 1:**
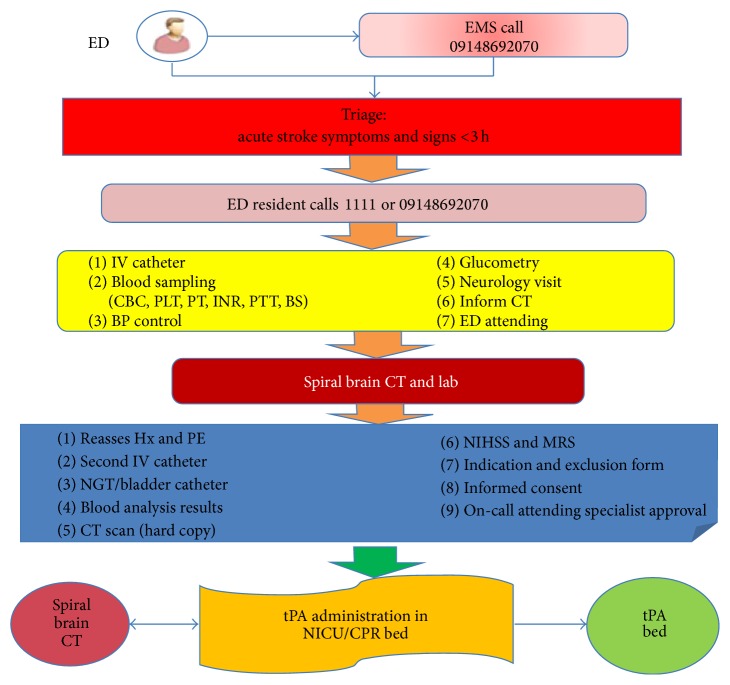
Management of acute ischemic stroke patients for tPA therapy in Tabriz Imam Reza Hospital.

For clarifying what should be done step by step when stroke code is being activated, an algorithm was made and explained during education sessions (Figure 1). Aiming to find algorithm obstacles, a maneuver with a stroke patient was done just before starting the postintervention period (on April 7, 2012). At this maneuver, stroke code was activated after patient was admitted at ED, followed by CT scan, lab analysis, and transfer to TPA bed. The team members could reach the DTN time of 38 minutes. The interventions were as follows.

#### 3.3.1. “Single-Call Notification”

We decided to use a specific phone number for reducing the time interval between patient arrival at the hospital and first neurology visit. Initially, we used a wireless phone, but the device was not working well at some places in the hospital; so we decided to use a regular mobile phone number. All calls to this phone number were being diverted to the “on-call” vascular neurologist, who was responsible during his 24-hour shift. This phone number, as “stroke code activation number,” was introduced to every medical staff involved in treating stroke patients in the hospital and to all other hospitals of the province. So anyone with any patient with probable symptoms of stroke could directly contact the vascular neurologist for consultation and code activation. Our hospital is the only center in the province that provides 7/24 intravenous thrombolytic treatment for acute ischemic stroke patients.

#### 3.3.2. Priority in CT Scanning

In some occasions, patients waited too much for CT scan. So by achieving a CT priority, after activation of the stroke code, the CT room does not accept any new patient until CT is done for the code-activated stroke patient. Furthermore, with this achievement, at the very crowded hours of CT room, we had the opportunity to use the alternative CT scan that is normally reserved for admitted patients and not for patients being referred from ED.

#### 3.3.3. Lab Priority

Previously at ED, the results for platelet count, prothrombin time, partial thromboplastin time, international normalized ratio, and blood sugar were available in 2 hours; but after collaborating with laboratory manager, the samples of stroke patients received precedence, leading to availability of results within 20–30 minutes.

#### 3.3.4. ICU Bed

After thrombolysis treatment, patients need to be admitted in the stroke unit for at least 24 hours for close monitoring of vital signs and neurologic status. We did not have stroke unit in our center so we used our neurology Intensive Care Unit (ICU) and specified one bed as “TPA bed” which is allocated just for acute stroke patients who receive thrombolytic therapy. This was a major hurdle because, at times, we could not find empty bed for our patients and therefore losing the opportunity of treating the patient with IV r-TPA.

#### 3.3.5. Education

“Time is brain”: The importance of time for early treatment of stroke patients was emphasized through multiple sessions for all medical staff involved in the process, including ED and neuro-ICU nurses and CT and lab technicians of all hospitals. This was done to create a sense of urgency for acute stroke patients.

### 3.4. Study Outcomes

The primary outcome of the study was the median DTC time, during the two study periods described previously. Secondary outcomes were the percentage of patients with DTC time of less than 25 minutes and DTN time less than 60 and 30 minutes.

### 3.5. Statistical Analysis

All statistical analyses were carried out using PASW statistics version 18. Due to nonnormal distribution of variables (i.e., age, DTC time, and DTN time), data are presented as median and interquartile range. Mann-Whitney *U* test was used to assess the mean difference between the two groups. A *P* value less than 0.05 was considered statistically significant.

### 3.6. Ethical Consideration

This study was approved by the Ethic Committee of Tabriz University of Medical Sciences. Informed consent was obtained from all individual participants included in the study.

## 4. Results

Overall, 489 patients with probable stroke according to Cincinnati stroke scale were admitted to the emergency department during the preintervention and postintervention periods. Of this total, 276 patients enlisted in the postintervention period, of which 106 were treated with IV r-TPA because of an acute ischemic stroke.

Demographic parameters and outcome measurements are shown in Table 1. As can be seen, the median DTC time decreased significantly from 75 (52.5–105) minutes in the preintervention period to 20 (12–30) minutes in the postintervention group (*P* < 0.001). The proportion of patients with DTC time less than 25 minutes increased significantly, from 3.0% in the preintervention period to 66.7% in the postintervention period (*P* < 0.001).

At the postintervention period, out of 276 patients with stroke code, 106 (38%) patients were treated with IV thrombolytic therapy. The median (IQR) DTN time was 55 (40–73) minutes. The proportion of patients with DTN time less than 60 and 30 minutes was 62.4% and 11.8%, respectively. In the postintervention period, the rate of intravenous thrombolysis was 6.1%.

## 5. Discussion

In this quality-improvement study, the DTC time in intravenous thrombolysis for acute stroke patients was reduced drastically and DTN time reached less than 60 minutes in 62% of patients, due to implementation of simple interventions. We had two major barriers with the interventions; firstly our hospital is located in Tabriz metropolitan city (serving for approximately 3.5 million people), which is the main referral hospital for the province and a trauma center as well; any intervention we were going to implement must not interfere with the treatment of other patients. Secondly, as being in a developing country, any expensive intervention would not be accepted. Multiple previous studies have shown the effect of different interventions on reducing in-hospital delay for patients with acute ischemic stroke [10–14]. In some of these interventions, the CT room was relocated to ED, some used point-of-care lab, and some allocated one or two rooms in ED for acute stroke patients [7, 14–16]. However, these kinds of interventions were either complicated or expensive and were not feasible for our setting. We used less complicated and more practical interventions which every hospital even in low income countries can afford.

At postintervention period, forty-five patients had prehospital notification (PHN) but none in the preintervention period. We excluded all, since PHN effectively reduces the DTC and DTN times and this has been shown well in multiple studies previously [10, 17–19].

The achieved postintervention DTC and DTN time of 20 and 55 minutes are lower than the recommended 25 and 60 minutes by the National Institute of Neurological Disorders and Stroke (NINDS).

At the preintervention period thrombolysis was not available at our center for acute stroke patients and we could not determine the DTN time. However, at the postintervention period median DTN was 55 (40–73) minutes, showing that, with the present algorithm, an acceptable time interval can be achieved.

In the United States, in the Get With the Guidelines-Stroke Program with 1082 hospitals participating, only 26.6% of all treated patients had the DTN time of less than 30 minutes [6]. In 2010, the AHA/ASA began an initiative to assist hospitals to reduce DTN time. The goal of this initiative, called “Target: Stroke,” is to achieve a DTN time of less than 60 minutes in at least 50% of acute ischemic stroke patients [20]. In our study, 62.4% of patients in the postintervention group had DTN time of less than 60 minutes, which seems to be acceptable compared to this goal. Van Schaik et al., after a quality-improvement project, reported a median DTN time of 25 minutes. The percentage of patients who had been treated within 60 minutes was 94% [21]. Meretoja et al. reported a significant reduction in all stroke timelines after implementation of a series of interventions (12 measures) over the years. The median DTN time was reduced dramatically from 105 minutes in 1998 to 60 minutes in 2003 and to 20 minutes in 2011. This resulted in 94% of patients being currently treated within 60 minutes of arrival [15]. In our study, 62.4% of patients had been treated within 60 minutes of hospital arrival, which showed remarkable improvement after the implementation of interventions but is not comparable with recently published studies, and more interventions are needed to hasten the flow of acute stroke patients in the hospital.

Our study had limitations. Firstly, while our interventions were successful in reducing DTC time and reaching an acceptable DTN time, we do not know whether they actually could reduce DTN time in case we had r-TPA in preintervention period.

Secondly, based on our experience, we know there were patients with acute stroke symptoms presented to ED within 3 hours of onset and stroke code was not activated for them. So we do not exactly know how much our education was effective in proper triage and code activation for these patients.

Thirdly, there was a considerable delay between brain CT and IV thrombolysis in this study. We think the reason is that, based on our algorithm, after CT scan we needed to transfer patients to TPA bed which is located in two floors up and ask for written inform consent and the treating physician needed to wait for hard copy of brain CT scan. In future, for further shortening of the time between brain CT and IV thrombolysis we should change the algorithm so that we can start thrombolysis in the CT room.

In summary, our study confirms the effect of multiple and simple inexpensive systemic improvements on reducing in-hospital delay for acute ischemic stroke patients and shows that acceptable DTC and DTN times can be achieved in a hospital with limited facilities. Nevertheless, further studies with emphasis on clinical outcomes are warranted.

## Figures and Tables

**Table 1:** Demographic parameters and outcome measurements.

	Before intervention *N* = 213	After intervention *N* = 276	*P* value
Age (years), median (range)	70 (27–95)	63 (24–89)	<0.001
Men, *n* (%)	104 (48.8)	142 (52.0)	*P* = 0.485^*∗*^
Median door-to-CT time (IQR), minutes	75 (52.5–105)	20 (12–30)	<0.001^†^
Door-to-CT time < 25 minutes, %	3.3%	66.7%	<0.001^*∗*^
Median door-to-needle time (IQR), minutes	—	55 (40–73)	
Door-to-needle time < 60 min, %	—	62.4%	
Door-to-needle time < 30 min, %	—	11.8%	

^*∗*^Groups were compared by Chi Square test.

^†^Groups were compared by Mann-Whitney *U* test.
